# *Chronic Diseases in Canada* and *Preventing Chronic Disease* Copublishing on Health in Aboriginal Populations* *This article is part of a joint publication initiative between *Preventing Chronic Disease* and *Chronic Diseases in Canada*. *Preventing Chronic Disease* is the primary publisher, while *Chronic Diseases in Canada* is the secondary publisher.


**Published:** 2010-12-15

**Authors:** Howard Morrison, Samuel F. Posner

**Affiliations:** Chronic Diseases in Canada, Public Health Agency of Canada; Preventing Chronic Disease, Centers for Disease Control and Prevention

The January 2011 issue includes 6 papers that are copublished by *Chronic Diseases in Canada* (*CDIC*) and *Preventing Chronic Disease* (*PCD*). In this example of copublishing, each journal is the primary publisher of 3 of the papers and secondary publisher of the other 3. Copublication is uncommon among scientific journals; however, it does offer an opportunity for journals to reach a broader readership with information about areas of common interest. The International Committee of Medical Journal Editors identifies this model of publishing as appropriate when 2 journals with different readerships are publishing information about topics that may appeal to both.

The idea of copublishing a collection of articles was born of discussions between the 2 journals about how to improve quality, reach, and impact. Both journals have similar missions of publishing research and best practices in public health in the United States and Canada. The premise of copublishing is based on the understanding that public health research and practice are global, crossing the physical barriers of national borders. Public health professionals in Canada and the United States face many of the same challenges in developing and implementing programs to prevent and manage chronic diseases. Copublishing of scientific papers helps demonstrate how public health is a global issue and allows both journals to reach a broader audience interested in chronic disease prevention and control.

In March 2010, the opportunity to copublish became a reality when we identified a set of papers on chronic disease prevention and control among Canadian Aboriginal people.

Copublishing requires more coordination than when either journal publishes independently. Editing styles, publication schedules, and multilingual translations are just a few of the elements that need to be coordinated. We appreciate the efforts of the staff of both journals in collaborating effectively. This collection is translated into 3 common languages spoken in the United States and Canada — French, Spanish, and English — reflecting our effort to make the collection available to a broad range of researchers. The authors are key partners in the copublishing effort; without their agreement, this initiative would not have been possible.

The collection includes 4 original research papers as well as an editorial by Malcolm King, PhD, scientific director of the Canadian Institutes of Health Research's Institute of Aboriginal Peoples' Health and a member of the Mississaugas of the New Credit First Nation (Ontario). Dr King's career spans 30 years; he is responsible for advancing Aboriginal public health research in Canada and has a clear understanding of the role of social determinants of health in achieving overall health. It is an honor to have him pen the editorial that preceded this collection. This special issue is also preceded by our introduction, as editors in chief of the 2 journals. Two of the 4 original research papers (Bruce et al and Riediger et al) are companion studies that involve a Manitoba First Nation community. Bruce et al looked at obesity in this population and the related comorbidities — dyslipidemia, hypertension, and diabetes. The other 2 papers (Ng and Tjepkema) are national studies. Ng and colleagues studied arthritis in the Canadian Aboriginal population, focusing on the differences between Canada's northern territories and the 10 provinces to the south. Tjepkema compared mortality patterns and rates among the urban-dwelling Canadian Aboriginal population and other urban residents.

These papers report data from Canadian Aboriginal people, but others have reported similar findings among US American Indian/Alaska Native people. These and other studies show a substantial burden of risk factors and chronic diseases in these populations. The articles document the need for public health interventions to address chronic disease prevention and management. Ng and colleagues note regional differences in arthritis. This finding is an example of the importance of recognizing that Aboriginal people are not a monolithic population and that different groups will require different interventions. Moving forward, development and evaluation of interventions are needed. As with any other high-risk group, documenting the burden is insufficient. Action to address the issues is where public health will improve the well-being of the populations it serves.

This landmark copublishing effort is a first for both *PCD* and *CDIC* and an innovation in the world of scholarly publication. With this effort, we are able to reach a broader range of researchers. Our initiative has the power to facilitate information sharing and discussion among researchers in this field. We hope that the readership of both journals will find joint publication useful and that it will be a model for further publishing efforts. In addition to the scientific discussion, we are interested in feedback on copublishing.

